# High glucose exacerbates neuroinflammation and apoptosis at the intermediate stage after post-traumatic brain injury

**DOI:** 10.18632/aging.203136

**Published:** 2021-06-27

**Authors:** Wenqian Zhang, Jun Hong, Wencheng Zheng, Aijun Liu, Ying Yang

**Affiliations:** 1 Department of Neurosurgery, Tangshan Gongren Hospital, Tangshan, Hebei 063000, China; 2 Hebei Institute of Head Trauma, Tangshan Gongren Hospital, Tangshan, Hebei 063000, China; 3 Department of Cardiology, Tangshan Gongren Hospital, Tangshan, Hebei 063000, China; 4 Department of Endocrinology, Tangshan Gongren Hospital, Tangshan, Hebei 063000, China

**Keywords:** high glucose, traumatic brain injury, neuroinflammation, apoptosis

## Abstract

Traumatic brain injury (TBI) is a highly lethal event with a poor prognosis. Recovering residual neuronal function in the intermediate stage of TBI is important for treatment; however, neuroinflammation and neuronal apoptosis impede residual neuronal repair processes. Considering that hyperglycemia influences inflammatory processes and neuronal survival, we examined the effects of high glucose on neuroinflammation and neuronal death during the intermediate phase of TBI. Rat models of type 2 diabetes mellitus and/or TBI were developed and behaviorally assessed. Neurological function and cognitive abilities were impaired in TBI rats and worsened by type 2 diabetes mellitus. Histopathological staining and analyses of serum and hippocampal mRNA and protein levels indicated that neuroinflammation and apoptosis were induced in TBI rats and exacerbated by hyperglycemia. Hyperglycemia inhibited hippocampal mitogen-activated protein kinase kinase 5 (MEK5) phosphorylation in TBI rats. *In vitro* assays were used to assess inflammatory factor expression, apoptotic protein levels and neuronal survival after MEK5 activation in TBI- and/or high-glucose-treated neurons. MEK5/extracellular signal-regulated kinase 5 (ERK5) pathway activation reduced the inflammation, cleaved caspase-3 expression, Bax/Bcl-2 ratio and apoptosis of TBI neurons, even under high-glucose conditions. Thus, high glucose exacerbated neuroinflammation and apoptosis in the intermediate stage post-TBI by inhibiting the MEK5/ERK5 pathway.

## INTRODUCTION

Traumatic brain injury (TBI) is a serious public health problem that poses heavy economic and societal burdens due to the high rates of death and disability among patients. An epidemiological investigation suggested that over 50 million people worldwide suffer from TBI and its associated sequelae, and that approximately half of the world population may have at least one TBI during their lifetime [1]. In addition to its direct mechanical effects, TBI induces a cascade of reactions that expand the tissue injury [2, 3]. This secondary damage involves physiological and pathological processes such as oxidative stress, autophagy, inflammation, neuronal survival pathways, etc. [3–5].

The early phase of TBI usually takes place within 24 h of injury, while the intermediate phase occurs in the initial days post-TBI and the late phase arises days to weeks after TBI. The pathophysiological processes in the early phase mainly result from energy expenditure and cell death due to excitotoxicity, whereas the intermediate stage is characterized by neuroinflammation and the late phase is marked by increased susceptibility [6]. After cerebral traumatic events, inflammatory reactions can serve as defense mechanisms against injuries, pathogens and toxins [7], but excessive inflammatory responses can increase the risk of brain tissue damage [8]. Thus, a proper balance must be maintained between pro- and anti-inflammatory responses to promote the survival and normal functioning of neural cells [9].

Clinical and animal studies have indicated that hyperglycemia is associated with poor outcomes in TBI patients [10–12]. Moreover, type 2 diabetes mellitus (DM) has been reported as a risk factor for mortality after a TBI event [13]. Among trauma intensive care unit patients, those with low blood glucose levels (4.4 to 6.1 mmol/L) appear to have lower morbidity and mortality than those with high blood glucose concentrations (exceeding 12 mmol/L), and low glucose levels have been associated with reduced incidences of critical illness polyneuropathy, bacteremia, acute renal failure, etc. [14]. High glucose (HG) has been reported to amplify the brain contusion volume and suppress neutrophil and macrophage infiltration on post-injury days 1–90, thus exacerbating neurological functional deterioration and inducing apoptosis [10]. However, other studies have shown that modestly high blood glucose allows neuronal cells to acquire sufficient energy to prevent secondary brain damage [12, 14]. Thus, the influence of hyperglycemia on inflammation and programmed cell death in the post-trauma period, especially the intermediate stage, is not clear at this point.

HG contributes to various inflammatory and apoptotic processes by altering numerous pathways, thus enabling cells to react to environmental changes [15, 16]. The effects of HG on cell survival and inflammatory cascades may be due to the mitogen-activated protein kinase kinase 5 (MEK5)/extracellular signal-regulated kinase 5 (ERK5) pathway inhibition, which downregulates pro-inflammatory factors and cell cycle-related proteins to prevent microglial polarization and β-cell apoptosis [17, 18]. There is evidence that ERK5 is activated by the upstream kinase MEK5 in response to diverse stimuli, including HG [17]. Upon phosphorylation by MEK5, ERK5 translocate to the nucleus to suppress genes involved in proinflammatory and apoptotic processes [17, 19].

Although hyperglycemia has been confirmed to regulate responses to multiple damaging stimuli, to the best of our knowledge, its effects during the inflammatory phase of TBI have not been examined. Thus, in the present study, we investigated the contribution of HG to inflammatory reactions and neuronal death during the intermediate phase of TBI, and explored the possible signaling pathways involved.

## RESULTS

### Hyperglycemia exacerbated neurological dysfunction and cognitive deficits

In this study, we established rat models of type 2 DM and TBI. After the DM rats had exhibited stable hyperglycemia for two weeks, 30 DM rats and 30 non-DM rats were used to establish the TBI model, while additional non-DM rats were subjected to a Sham TBI operation. Thus, four groups of rats were analyzed: Sham, DM, TBI and DM+TBI.

**Figure 1:**
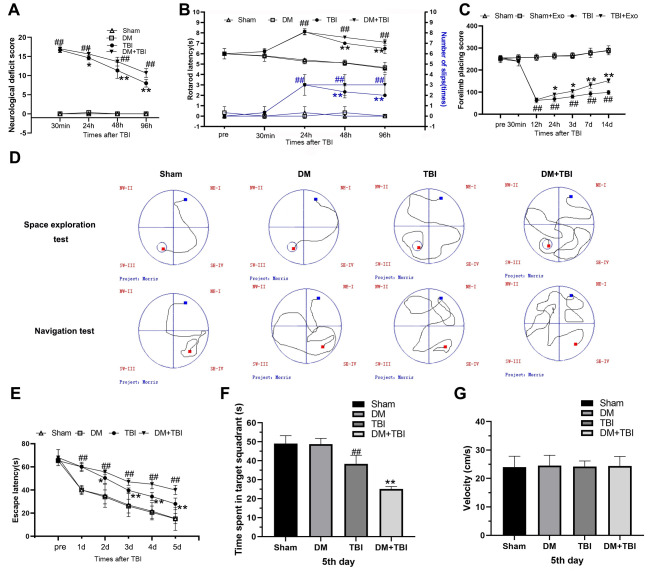
**Hyperglycemia exacerbated neurological dysfunction and cognitive deficits due to TBI.** (**A**) mNSS. (**B**) Rotating rod test. (**C**) Forelimb placement test. (**D**–**G**) MWM test. All data are presented as the mean ± standard error (*n* = 8 per group). Statistical significance was determined using one-way ANOVA followed by *post-hoc* Bonferroni correction. ^#^*P* < 0.05 or ^##^*P* < 0.01 vs. the Sham group; ^*^P < 0.05 or ^**^P < 0.01 vs. the TBI group.

Behavioral performance was evaluated using the modified neurological severity score (mNSS), rotarod test and forelimb placement test. Varying degrees of behavioral defects were observed in the TBI rats (Figure 1A). The rats in the DM+TBI group exhibited poorer mNSS performance than those in the TBI group over time (24, 48 and 96 h). Notably, hyperglycemia alone did not impair the rats’ neurological abilities (*P* > 0.05). The rotarod latencies of the TBI rats were significantly shorter than those of the Sham rats (*P* < 0.01; Figure 1B). Moreover, hyperglycemia significantly reduced the rotarod latencies of the TBI rats over time (*P* < 0.01). As for the forelimb placement test, TBI rats exhibited significant forelimb placement deficits (*P* < 0.01), and HG aggravated these deficits in TBI rats (*P* < 0.05; Figure 1C).

We also tested learning and memory using Morris Water Maze (MWM) positioning navigation and space exploration tests. In the positioning navigation test, the escape latency was prolonged after the onset of TBI (*P* < 0.01; Figure 1D and 1E), and this effect was even more pronounced in the DM+TBI group. In the space exploration test, the TBI rats exhibited significantly poorer performance than the Sham rats after the platform was removed, and a further decrease in the target quadrant time was observed in the DM+TBI group compared with the TBI group (*P* < 0.01). In both the positioning navigation and space exploration tests, DM alone did not induce notable learning or memory deficits (both *P* > 0.05; Figure 1E and 1F). As illustrated in Figure 1G, there were no significant differences in swimming speed throughout the trials (*P* > 0.05).

### HG aggravated the pathological damage caused by TBI

**Figure 2:**
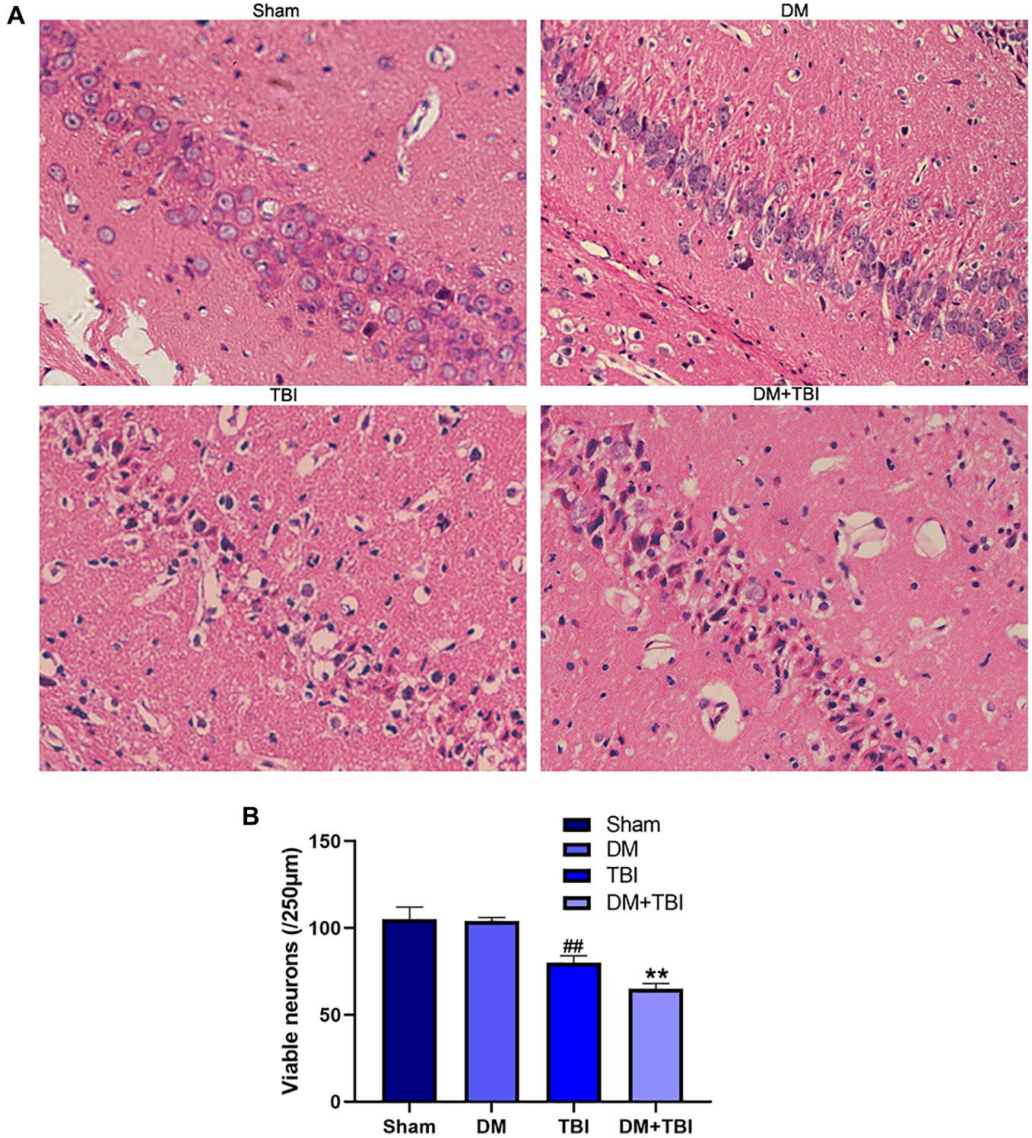
**HG aggravated pathological injuries caused by TBI.** (**A**) Hematoxylin and eosin staining (scale bar, 20 μm). (**B**) Quantification of the number of viable neurons per 250-lm length in each group. All data are presented as the mean ± standard error (*n* = 4 per group). Statistical significance was determined using one-way ANOVA followed by *post-hoc* Bonferroni correction. ^##^*P* < 0.01 vs. the Sham group; ^**^P < 0.01 vs. the TBI group.

Next, we used hematoxylin and eosin staining to detect morphological alterations in hippocampal neurons (Figure 2). Hippocampal tissues from the Sham group exhibited normal morphological characteristics, including round nuclei and clear cytoplasms. Likewise, no significant neuronal injury was found in the DM group. However, neurons from the TBI group exhibited various histopathological changes, including cell loss, nuclear pyknosis and neuronal atrophy. HG worsened the neuronal cell loss and atrophy of TBI rats.

### Hyperglycemia increased pro-inflammatory and reduced anti-inflammatory factor levels

**Figure 3:**
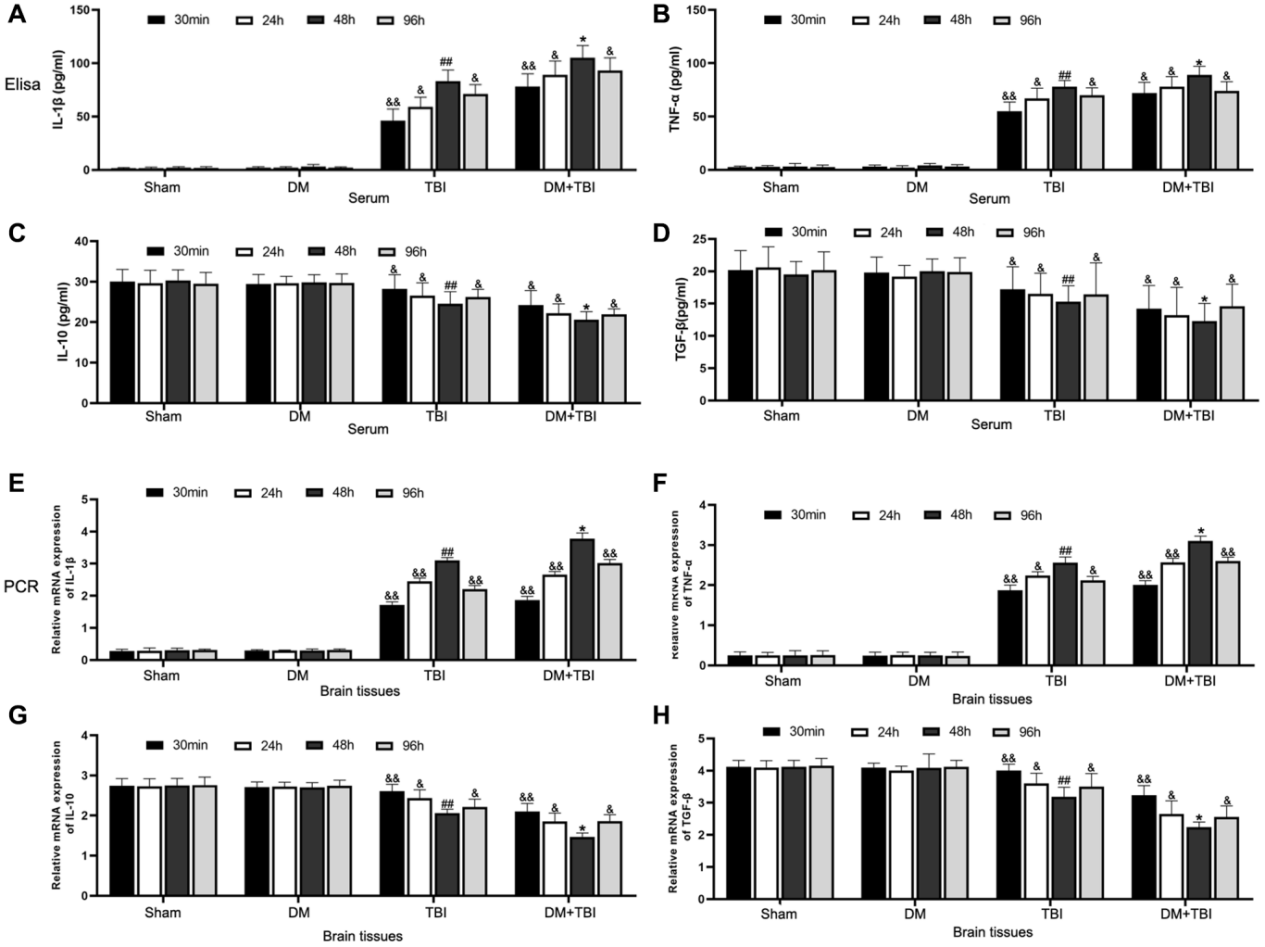
**Hyperglycemia upregulated pro-inflammatory and downregulated anti-inflammatory factors.** (**A**) Serum IL-1β. (**B**) Serum TNF-α. (**C**) Serum IL-10. (**D**) Serum TGF-β. (**E**) Hippocampal *IL-1β* mRNA. (**F**) Hippocampal *TNF-α* mRNA. (**G**) Hippocampal *IL-10* mRNA. (**H**) Hippocampal *TGF-β* mRNA. *β-actin* mRNA expression was used as the qRT-PCR control. All data are presented as the mean ± standard error (*n* = 8 per group). Statistical significance was determined using one-way ANOVA followed by *post-hoc* Bonferroni correction. ^&^*P* < 0.05 or ^&&^*P* < 0.01 vs. results at 48 h; ^#^*P* < 0.05 or ^##^*P* < 0.01 vs. the Sham group; ^*^P < 0.05 or ^**^P < 0.01 vs. the TBI group.

We then performed enzyme-linked immunosorbent assays to measure the levels of pro- and anti-inflammatory factors in serum samples from the different groups of rats. Serum interleukin (IL)-1β and tumor necrosis factor alpha (TNF-α) levels were significantly greater in TBI rats than in Sham rats (*P* < 0.01; Figure 3A and 3B), whereas serum IL-10 and transforming growth factor beta (TGF-β) levels were lower in TBI rats than in Sham rats (Figure 3C and 3D). This inflammatory reaction was most prominent 48 h after TBI. Hyperglycemia did not alter the inflammatory states of non-TBI rats. However, compared with TBI rats, pro-inflammatory factor (IL-1β and TNF-α) levels rapidly increased and anti-inflammatory factor (IL-10 and TGF-β) levels significantly decreased when DM rats underwent TBI (*P* < 0.05).

We also performed quantitative real-time PCR (qRT-PCR) to examine the same pro- and anti-inflammatory factors in hippocampal tissues from the rats (Figure 3E–3H). Mild inflammatory responses were observed in hippocampal tissues from Sham and DM rats; however, pronounced inflammatory responses and weak anti-inflammatory responses were detected in hippocampal tissues from TBI rats. Hyperglycemia further induced the inflammatory response in TBI rats, as evidenced by increased pro-inflammatory and reduced anti-inflammatory factor expression.

### Hyperglycemia inhibited MEK5 phosphorylation and MEK5/ERK5 pathway activation in TBI rats

**Figure 4:**
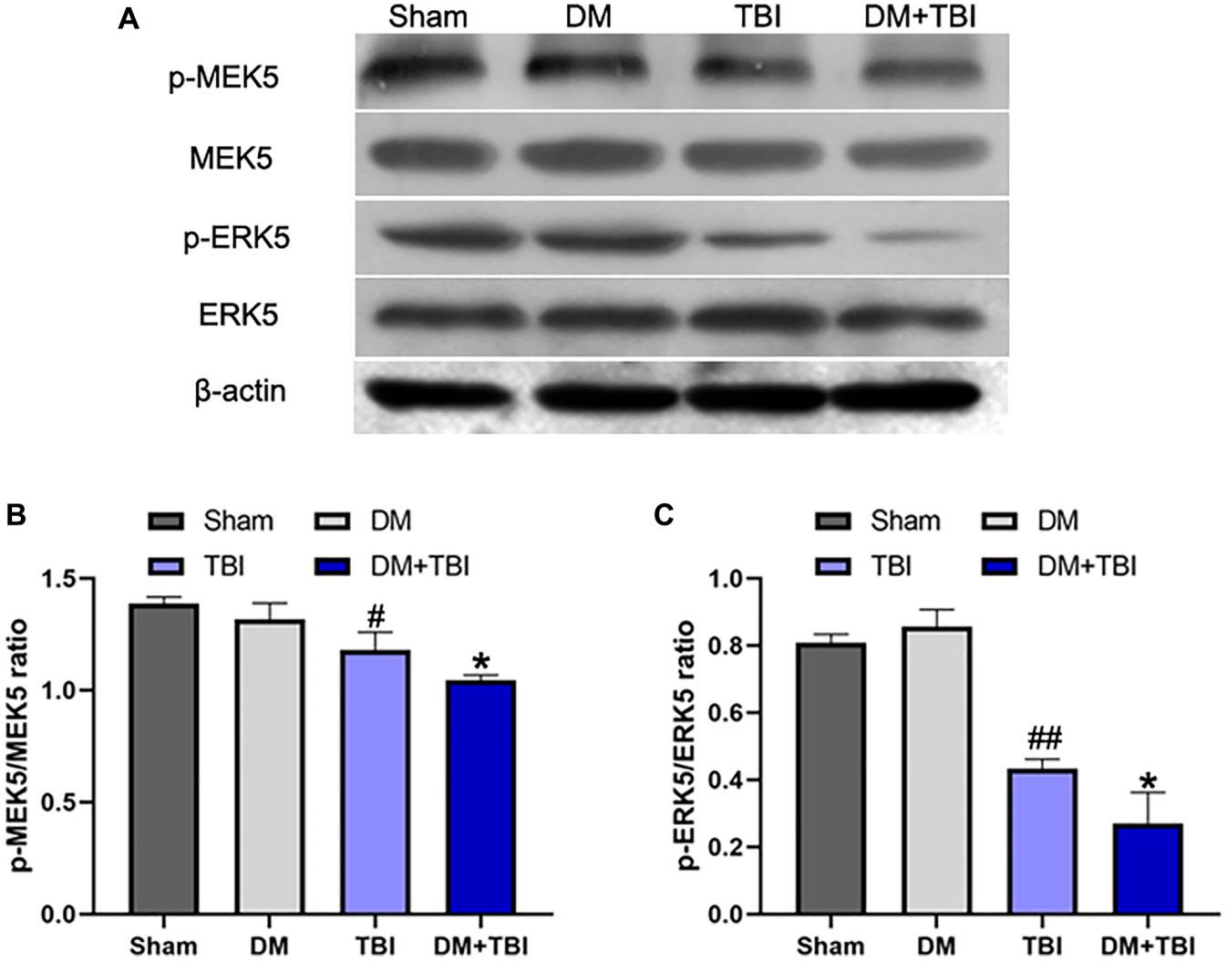
**Hyperglycemia inhibited MEK5 phosphorylation and MEK5/ERK5 pathway activation.** (**A**) Western blot analysis of MEK5/ERK5 protein phosphorylation in the hippocampus 48 h after TBI or sham surgery. Bar graphs illustrate densitometric analyses of (**B**) p-MEK5/MEK5 and (**C**) p-ERK5/ERK5. All data are presented as the mean ± standard error (*n* = 5 per group). Statistical significance was determined using one-way ANOVA followed by *post-hoc* Bonferroni correction. ^#^*P* < 0.05 or ^##^*P* < 0.01 vs. the Sham group; ^*^P < 0.05 or ^**^P < 0.01 vs. the TBI group.

Next, we examined the phosphorylation (activation) of MEK5 and its downstream target ERK5 in hippocampal tissues from the various groups. Phosphorylated (p)-MEK5 and p-ERK5 levels in hippocampal tissues were lower in the TBI group than in the Sham group (*P* < 0.01). Moreover, MEK5 phosphorylation was reduced and MEK5/ERK5 pathway activation was dramatically inhibited in the DM+TBI group relative to the TBI group (*P* < 0.05; Figure 4). However, hyperglycemia did not induce this effect in non-TBI rats (*P* > 0.05).

### HG exacerbated neuronal apoptosis after TBI

**Figure 5:**
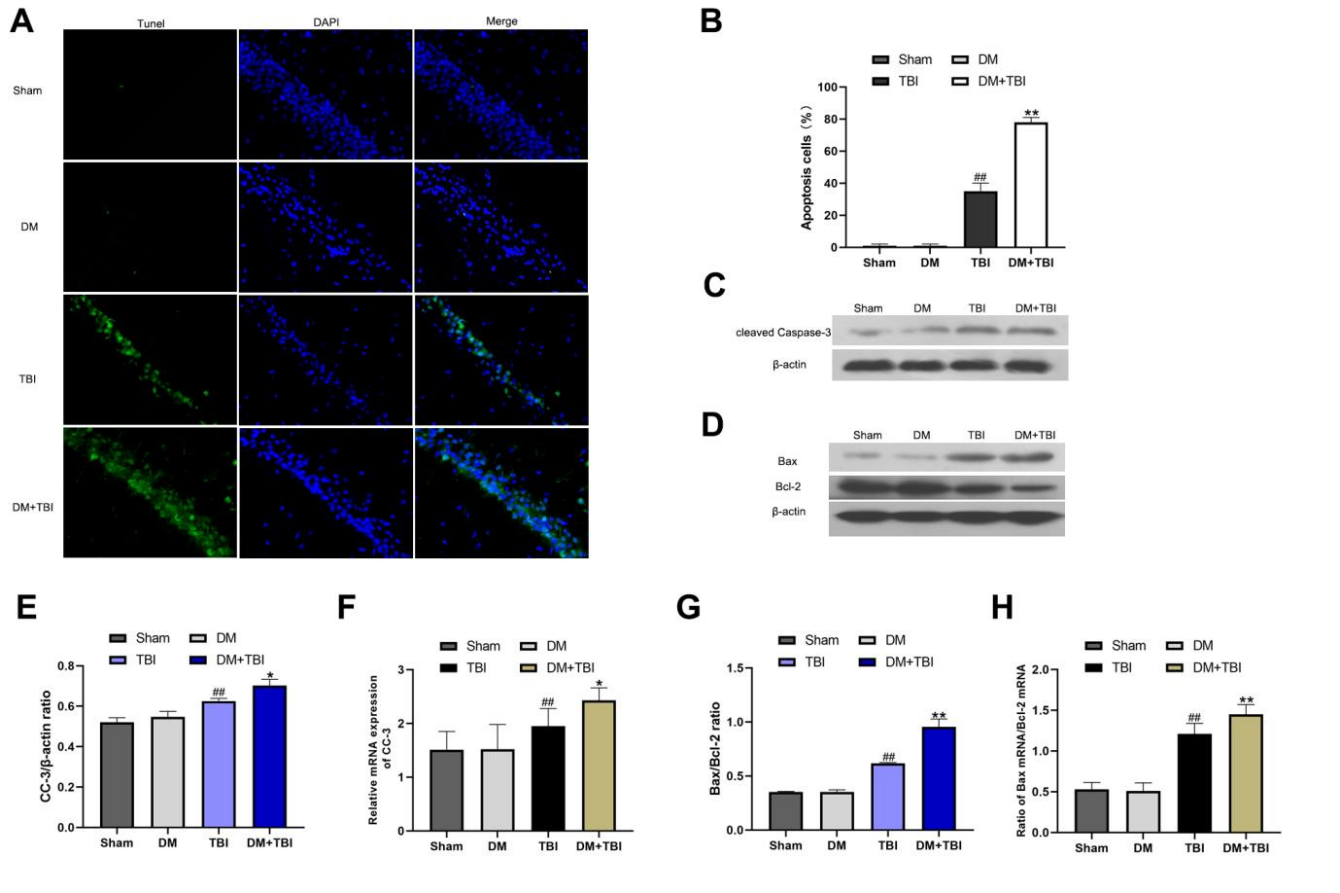
**HG exacerbated neuronal apoptosis after TBI. Apoptosis was assessed using DAPI and TUNEL staining 48 h after TBI (scale bar, 50 μm).** (**A**) Representative confocal images of hippocampal tissues stained with TUNEL (green) and DAPI (blue). (**B**) Bar graph of the proportion of apoptotic cells. Western blot analysis of (**C**) CC-3 and (**D**) Bax/Bcl-2 protein levels in the hippocampus 48 h after TBI or sham surgery. Bar graphs illustrate densitometric analyses of the Western blot protein bands for (**E**) CC-3 and (**G**) Bax/Bcl-2, each normalized to β-actin. Bar graphs illustrate quantitative analyses of (**F**) *CC-3* and (**H**) *Bax/Bcl-2* mRNA levels, each normalized to *β-actin*. All data are presented as the mean ± standard error (*n* = 5 per group). Statistical significance was determined using one-way ANOVA followed by *post-hoc* Bonferroni correction. ^#^*P* < 0.05 or ^##^*P* < 0.01 vs. the Sham group; ^*^P < 0.05 or ^**^P < 0.01 vs. the TBI group.

We then assessed neuronal apoptosis in the hippocampus using terminal deoxynucleotidyl transferase dUTP nick end labeling (TUNEL) staining. The proportion of TUNEL-positive cells was significantly greater in the TBI group than in the Sham group (Figure 5A and 5B). Moreover, the proportion of apoptotic neurons was markedly induced in the DM+TBI group compared with the TBI group (*P* < 0.01).

To further explore the effects of HG and TBI on neuronal apoptosis in the hippocampus, we used Western blotting and qRT-PCR to detect the expression of cleaved caspase-3 (CC-3), Bax and Bcl-2. In the Western blot analysis, CC-3 expression and the Bax/Bcl-2 ratio in hippocampal tissues were noticeably greater in TBI rats than in Sham rats (*P* < 0.01; Figure 5C–5E, 5G). The CC-3 level and Bax/Bcl-2 ratio was higher in the DM+TBI group than in the TBI group, indicating that preexisting DM exacerbated hippocampal neuronal apoptosis in TBI rats. Similar trends were observed in the qRT-PCR experiments (Figure 5F and 5H).

### HG significantly inhibited scratched cell viability

**Figure 6:**
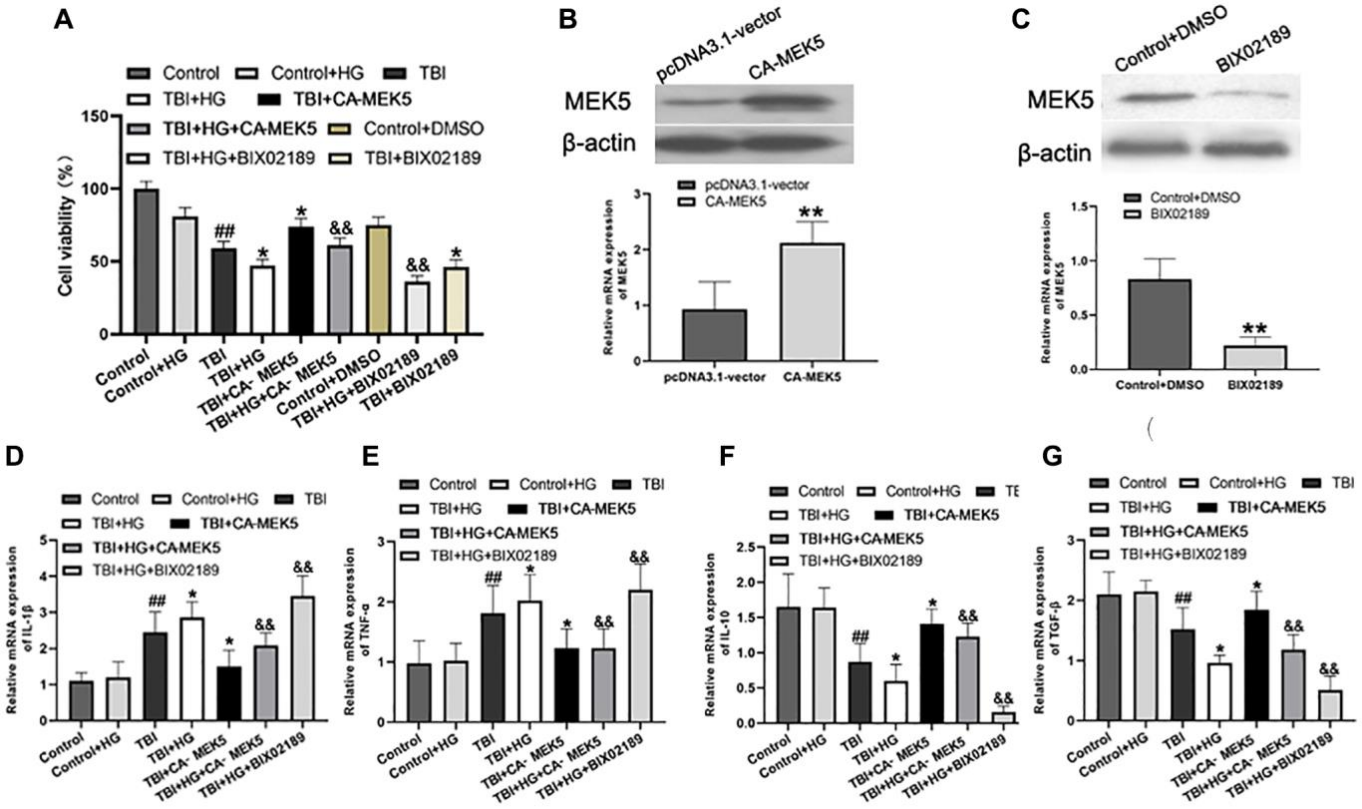
**HG reduced cell viability and aggravated neuroinflammation by inhibiting the MEK5/ERK5 pathway.** (**A**) Cellular viability was detected using a CCK-8 assay. The transfection efficiency was confirmed using (**B**) Western blot analysis and (**C**) qRT-PCR. ^**^P < 0.01 vs. the pcDNA3.1 vector group. Bar graphs illustrate the relative mRNA levels of (**D**) *IL-1β*, (**E**) *TNF-α*, (**F**) *IL-10* and (**G**) *TGF-β*. *β-actin* was used as the qRT-PCR control. Data are presented as the mean ± standard deviation (*n* = 5 per group). Statistical significance was determined using one-way ANOVA followed by *post-hoc* Bonferroni correction. ^#^*P* < 0.05 or ^##^*P* < 0.01 vs. the Control group; ^*^P < 0.05 or ^**^P < 0.01 vs. the TBI group; ^&^*P* < 0.05 or ^&&^*P* < 0.01 vs. the TBI+HG group.

Next, we generated an *in vitro* model of TBI by scratching monolayers of primary rat hippocampal neurons with a pipette tip. Certain groups of cells were subjected to HG conditions and/or transfected with a constitutively active rat MEK5 mutant plasmid (CA-MEK5). In total, six groups were examined: Control, Control+HG, TBI, TBI+HG, TBI+CA-MEK5 and TBI+HG+CA-MEK5. Western blotting and qRT-PCR were used to confirm that MEK5 was overexpressed in the CA-MEK5-treated cells (Figure 6B and 6C).

Subsequently, a Cell Counting Kit 8 (CCK-8) assay was used to assess cell viability. As shown in Figure 6A, exposure of unstimulated primary neurons to HG did not alter their viability, whereas scratched neurons exhibited low viability in normal-glucose medium. Remarkably, scratched neurons subjected to HG conditions (TBI+HG) were noticeably less viable than TBI neurons after two days. The neurons in the TBI+HG+CA-MEK5 group displayed much higher viability than those in the TBI+HG group (*P* < 0.05), although they did not reach the viability of the TBI+CA-MEK5 group (*P* < 0.05).

### HG aggravated neuroinflammation by inhibiting the MEK5/ERK5 pathway

We then performed qRT-PCR to assess whether HG or TBI conditions induced inflammatory responses in neurons *in vitro*, and whether these effects were due to the inactivation of MEK5/ERK5. Primary hippocampal neurons cultured in HG medium exhibited slightly greater inflammation than Control neurons, although the difference was not statistically significant (Figure 6D–6G). Scratched neurons exhibited serious inflammation relative to Control+HG neurons, while TBI+HG neurons displayed the most severe inflammatory response. CA-MEK5 treatment significantly reversed the increase in *IL-1β* and *TNF-α* mRNA levels and the reduction in *IL-10* and *TGF-β* mRNA levels in the TBI+HG group (*P* < 0.01; Figure 6D–6G). However, cells in the TBI+HG+CA-MEK5 group exhibited more severe neuroinflammation than cells in the TBI+CA-MEK5 group.

### HG increased TBI-induced apoptosis by inhibiting the MEK5/ERK5 pathway

**Figure 7:**
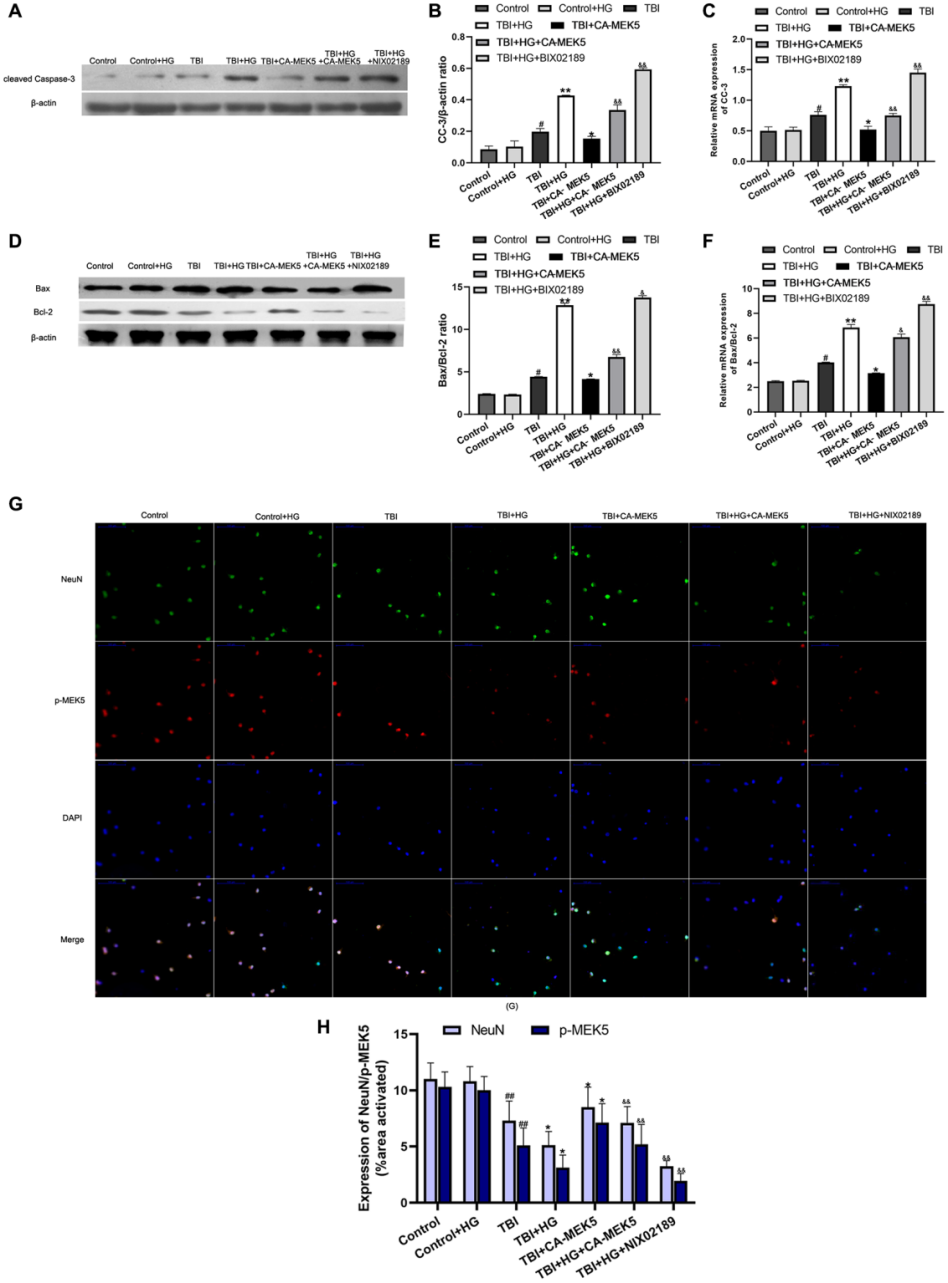
**HG increased TBI-induced apoptosis by inhibiting the MEK5/ERK5 pathway.** (**A**) CC-3 and (**D**) Bax/Bcl-2 protein bands in scratched and transfected primary hippocampal cells. Bar graphs illustrate densitometric analyses of the Western blot protein bands for (**B**) CC-3 and (**E**) Bax/Bcl-2, each normalized to β-actin. Bar graphs illustrate quantitative analyses of (**C**) *CC-3* and (**F**) *Bax/Bcl-2* mRNA levels, each normalized to *β-actin*. (**G**) Double immunofluorescent staining of NeuN and p-MEK5. Representative confocal images stained for p-MEK5 (red) and NeuN (green) demonstrate that CA-MEK5 treatment not only increased p-MEK5 protein levels, but also markedly increased neuronal survival (scale bar, 100 μm). (**H**) Staining for NeuN and p-MEK5 was analyzed using MATLAB software. Data are presented as the mean ± standard deviation (*n* = 5 per group). ^#^*P* < 0.05 vs. the Control group; ^*^P < 0.05 or ^**^P < 0.01 vs. the TBI group; ^&^*P* < 0.05 or ^&&^*P* < 0.01 vs. the TBI+HG group.

Finally, we investigated whether HG or TBI treatment induced neuronal apoptosis by suppressing MEK5/ERK5 *in vitro*. In Western blot analyses, the CC-3 level and Bax/Bcl-2 ratio in neurons did not differ between the Control+HG group and the Control group. The CC-3 protein level and Bax/Bcl-2 ratio were greater in the TBI group than in the Control+HG group (both *P* < 0.05; Figure 7A, 7B, 7D, 7E), and were even higher in the TBI+HG group. The CC-3 level and Bax/Bcl-2 ratio in TBI+HG neurons declined significantly following CA-MEK5 treatment (both *P* < 0.05), suggesting that HG exacerbated neuronal apoptosis by suppressing the MEK5/ERK5 pathway. However, apoptotic protein levels were greater in the TBI+HG+CA-MEK5 group than in the TBI+CA-MEK5 group. The mRNA levels of *CC-3* and the *Bax/Bcl-2* ratio followed the same trend as the protein levels (Figure 7C and 7F).

We also evaluated neuronal survival by performing double immunofluorescent staining with antibodies for p-MEK5 and neuronal nuclei (NeuN). The results indicated that HG not only reduced p-MEK5 protein expression in TBI neurons, but also significantly reduced the survival of these cells. However, the administration of CA-MEK5 increased hippocampal neuronal survival following TBI+HG treatment (*P* < 0.05; Figure 7G and 7H).

## DISCUSSION

According to an epidemiological study, over 50 million people suffer from TBI and its consequential conditions, and roughly half of the global population may experience a TBI at some point [1]. The pathology of TBI is usually divided into two phases: primary and secondary injuries [2]. Contemporary therapy should be designed to prevent secondary brain damage in TBI patients.

After the occurrence of trauma, residual neurons undergo significant functional alterations, and peripheral cells such as astrocytes and glial cells are recruited. Recruited astrocytes have been found to proliferate and secrete inflammatory mediators that damage neurons at injury sites, while glial cells have been found to induce phagocytosis and also secrete inflammatory mediators. These processes could be disturbed in HG environments [20, 21]. Indeed, HG microenvironments can induce regional acidosis [22, 23], oxidative stress responses and edema formation, and can activate inflammatory processes by promoting leukocyte infiltration [24].

In the present study, we observed that HG exacerbated hippocampal injury and exaggerated the neurological deficits and learning/memory problems after TBI. The negative effects of HG were most noticeable 48 h after TBI, possibly due to the inflammatory cascade. A previous study suggested that the inflammatory response in brain tissue was especially pronounced during the intermediate phase post-TBI [6]. In our rats, IL-1β and TNF-α levels increased in the first 48 h after TBI and tended to decline afterwards, while IL-10 and TGF-β levels decreased in the first 48 h after TBI and tended to increase afterwards, in agreement with an earlier study [25]. We found that HG reduced anti-inflammatory and increased pro-inflammatory cytokine expression, thus enhancing the inflammatory reaction in TBI rats. IL-10 and TGF-β have been reported to exert robust immunomodulatory and anti-inflammatory effects by inhibiting pro-inflammatory cytokine synthesis and suppressing cytokine receptor activation [26]. Our results may result from IL-10 and TGF-β secretion increased gradually with time after TBI, and HG did not change the trends obtained in cytokine assays, but did alter cytokine production.

Similar to our results, Bahniwal et al. [27] reported that HG increased the release of pro-inflammatory mediators from astrocytes, thereby contributing to neuronal injury. In another study, increasing the extracellular glucose concentration activated microglia, induced inflammation and worsened neural damage [28]. HG was recently reported to induce inflammation in human brain microvascular endothelial cells, thus compromising the blood-brain barrier [29]. In animal model of TBI in this study, the levels of all inflammation-related cells were found to increase post-TBI. HG can disturb the microcirculation and increase neutrophil infiltration [30]. Aggravated inflammatory reactions can exaggerate neuronal injury and apoptosis, ultimately worsening the prognosis of TBI. Thus, HG exacerbates inflammation-induced neural damage in a multifaceted manner.

Our results indicated that HG aggravated neuroinflammation partly by inhibiting the MEK5/ERK5 signaling pathway. In a previous study, reduced insulin sensitivity, elevated glucose levels and high Plasminogen Activator Inhibitor 1 levels (an inflammatory marker) were observed in adipose-specific ERK5-knockout mice [31]. ERK5 is a conventional mitogen-activated protein kinase (MAPK) subfamily member with multiple physiological and regulatory functions in diverse cell types [32, 33]. In a cascade of phosphorylation reactions, a MAPK kinase kinase phosphorylates MEK5, which ultimately phosphorylates ERK5, and activated ERK5 translocates to the nucleus to activate transcription [34]. MEK5 is the sole upstream MAPK kinase that activates ERK5, and MEK5 overexpression does not alter the activation of other MAPK family members.

MAPK and the nuclear fraction of nuclear factor-κB are the core components of the receptor signaling pathway involved in innate immune responses induced by inflammation [35]. ERK5 is an important contributor to inflammation and inflammation-driven cancer, as it regulates the release of several inflammatory factors and establishes the inflammatory microenvironment [36]. A previous study demonstrated that chronic hyperglycemia promoted microglial polarization into an increasingly pro-inflammatory subtype by inhibiting MEK5/ERK5 signaling [17]. Thus, the MEK5/ERK5 signaling pathway seems to be an important regulator of the inflammatory response and systemic glucose metabolism.

In addition to activating inflammation, HG has been reported to reduce neuronal survival by upregulating CC-3 and disrupting the balance between Bax and Bcl-2 [37]. The pro-apoptotic effects of HG have frequently been observed in individuals exposed to prolonged hyperglycemia [37–39]. ERK5 was found to inhibit endoplasmic reticulum stress and apoptosis in pancreatic β-cells, thereby ameliorating the tissue damage caused by hyperglycemia [40], and similar observations have been made in other cell types [41, 42]. In the present study, significant apoptosis was observed among scratched hippocampal primary neurons, and HG worsened this effect while also downregulating the MEK5/ERK5 pathway. Treatment with CA-MEK5 significantly reduced neuronal apoptosis; however, TBI+HG+CA-MEK5 neurons still exhibited lower p-MEK5 expression and greater apoptosis than TBI+CA-MEK5 neurons. Thus, good glycemic control helps to attenuate hippocampal neuronal damage due to the inflammatory response and apoptosis. Notably, hypoglycemia has been documented to induce glutamate release, thereby impairing metabolism [21]. Therefore, it is particularly important to maintain blood glucose within a proper range to prevent irreversible central nervous system lesions.

A previous study indicated that the immune system was dysregulated in patients with type 2 DM [43]. This loss of homeostatic control may contribute to tissue-specific and systemic inflammation and immune alterations. However, hyperglycemia has exhibited different effects on the immune response in different studies [44]. Thus, the involvement of HG in TBI-dependent immune responses needs to be further investigated. In addition, activation of the MEK5/ERK5 pathway has been reported to gradually increase tumor risk [45]. Therefore, further research is required to develop therapeutic interventions that reduce the mortality rate of TBI patients without increasing tumor risk.

**Figure 8:**
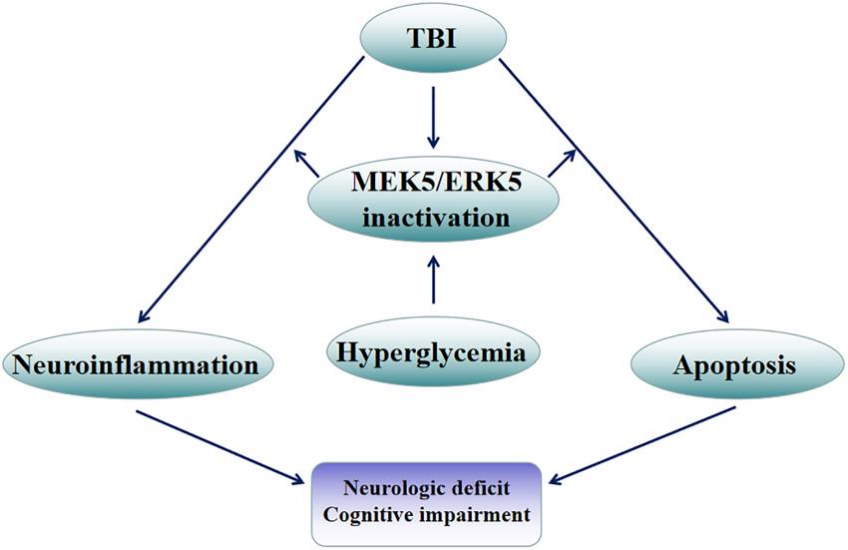
**Mechanistic diagram.** HG exacerbated hippocampal injury following TBI, largely by disrupting the balance between pro- and anti-inflammatory factors and inducing apoptosis in hippocampal neurons. The MEK5/ERK5 pathway was found to participate in both of these processes.

In conclusion, HG exacerbated hippocampal injury after TBI, largely by disrupting the homeostasis of pro- and anti-inflammatory cytokines and inducing hippocampal neuronal apoptosis. The MEK5/ERK5 pathway participated in both of these processes (Figure 8). These experimental findings emphasize the importance of good glycemic control for the functional recovery of residual neurons in TBI patients.

## MATERIALS AND METHODS

### TBI and type 2 DM models

The animal experiments in this study were conducted according to the National Institutes of Health Guide for the Care and Use of Laboratory Animals [46]. The study was approved by the Ethics Committee of Tangshan Gongren Hospital (approval no. GRYY-LL-2020-100). For all animal experiments, adult male Sprague-Dawley rats (age 10–12 weeks; weight 210–260 g; 120 rats in total) were obtained from the Experimental Animal Center of North China University of Science and Technology. The rats had free access to food and water throughout the trial. The housing conditions were as follows: 20–23°C, 12 h light/12 h dark and 50 ± 10% humidity. The rats were randomly divided into four groups (Sham, DM, TBI and DM+TBI), with 29–30 rats in each group. During the modeling, one rat died in the DM group and one rat died in the TBI group.

Type 2 DM was induced through high-fat diet feeding and streptozotocin injection, which simulates the natural disease progression and metabolic characteristics of type 2 DM. The high-fat diet contained 60% kcal from fat (Research Diets, NJ, USA). A random-number table was used to select 60 rats for the rat model of type 2 DM. After two months of high-fat diet feeding, the successful induction of insulin resistance was determined using an intraperitoneal glucose tolerance test. Streptozotocin was dissolved in 0.1 mol/L sodium citrate buffer (pH 4.4), and was injected intraperitoneally into the rats (60 mg/kg) for five days. The rats in the Sham group were fed a normal diet containing 10% kcal from fat, and were intraperitoneally injected with an equal amount of citrate buffer. After five days of streptozotocin administration, the rats were fasted for 3 h, and tail vein blood was collected for blood glucose analysis. Successful DM model rats were defined as those with fasting blood glucose levels ≥ 250 mg/dL (13.9 mmol/L).

The TBI model was generated according to a previously reported method [47]. Briefly, after the DM rats had exhibited stable hyperglycemia for two weeks, 30 DM rats and 30 non-DM rats were randomly chosen to establish the TBI model. Each rat was anesthetized with an intraperitoneal injection of sodium pentobarbital (50 mg/kg). The head of the rat was fixed on a stereotactic frame, and an incision was made in the scalp along the midline of the skull. Centered on the coronal suture, a 6-mm craniotomy was made ~2.5 mm lateral to the sagittal suture above the right parietal cortex. TBI was induced using a strike depth of 2.5 mm, a dwell time of 100 ms and an impact velocity of 5 m/s. Afterwards, the bone flap was immediately replaced and sealed, and the scalp was closed with sutures. The rectal temperature of the rat was maintained at 37–37.5°C with a heating pad throughout the surgery. After waking from anesthesia, the rat was returned to its home cage. The Sham group underwent the anesthesia, craniotomy and bone flap replacement without the TBI. All experiments were performed under sterile conditions.

### Neurological functional assessment

Neurological function was assessed 30 min, 24 h, 48 h and 96 h after TBI. The mNSS was assigned as previously described [48], based on sensory, motor, reflex and beam balance tests. Higher scores indicated worse neural defects (least severe = 0; most severe =18).

### Rotarod performance and forelimb placement tests

Rotating rod and forelimb placement tests were used to assess the motor functioning and neurological outcomes of the rats. The rats were tested before TBI and 30 min, 24 h, 48 h and 96 h after TBI. For the rotating rod test, the rat was placed on an accelerating automated rotarod with a rotation speed of 5–40 rpm throughout the trial, in accordance with our previously reported method [48]. As the rotation speed increased, the average time until the rat fell twice from the rotating drum was recorded. Two independent observers recorded all results manually. Each rat performed three trials per day.

The forelimb placement test method has been reported previously [49]. In brief, experienced testers who were blinded to the condition of the rats performed the tests. The rat was held by its torso so that its forelimbs hung free. Before the test, the rat was gently moved upward to relax its muscles. Data were excluded if the rat struggled, exhibited extreme muscle tension or placed any of its limbs on the experimenter’s hand. Each forelimb was tested independently. The respective vibrissae were brushed on the corner edge of a countertop, under which conditions a healthy rat would promptly place the forelimb ipsilateral to the stimulated vibrissae onto the countertop. Each rat was tested 10 times for each forelimb, and the percentage of times that the rat successfully placed the appropriate forelimb on the tabletop was determined.

### MWM test

MWM tests were performed to evaluate memory retention and learning abilities during days 1–5 after TBI. We used a previously described procedure and water maze apparatus [37]. The trials were carried out at a fixed time each training day. Before the start of the positioning navigation test, the rat was placed in a round water tank and allowed to swim freely for 5 min. A hidden platform was placed horizontally at the center of the target quadrant. The rat was placed inside the tank in any of the other three quadrants not containing the platform, and was given time to locate the hidden platform. Any rat that found the platform was placed on the platform for an additional 15 seconds. Any rat that could not find the platform or failed to escape within 90 seconds was guided to the platform and allowed to stand for 15 seconds, and the measurement was recorded as 90 seconds. Each rat was tested four times per day for four days.

For the space exploration test, the platform was removed and the rat was permitted to swim in the water for 60 seconds on the fifth day of the experiment. The time the rat spent in the target quadrant was recorded. A video camera interfaced with a video tracking system (HVS Image Software Ltd., Hampton, UK) was placed above the tank to record and analyze the performance.

### Hematoxylin and eosin staining

At different time points after the behavioral experiments, the rats were anesthetized and perfused transcardially. The rats were then sacrificed via decapitation, and the hemispheres of their brains were rapidly removed. For hematoxylin and eosin staining, samples were obtained 48 h after TBI. Seven days after being fixed with 5% neutral buffered formalin at room temperature, the brain tissues were sliced into 4-μm sections. The sections were stained with hematoxylin for 2 min and with eosin for 30 seconds. Histological changes in hippocampal regions were visualized using an optical microscope (Olympus Corporation, Tokyo, Japan; 200× magnification). The number of neurons per 250-lm length of the CA1 pyramidal cell layer was counted in five sections per rat, and the average was taken as the result.

### Detection of pro- and anti-inflammatory factors

Blood samples were collected from rats in each group 24, 48 and 96 h after TBI. For *in vitro* experiments, cell supernatants were collected. The concentrations of pro-inflammatory factors (IL-1β and TNF-α) and anti-inflammatory factors (IL-10 and TGF-β) were measured using enzyme-linked immunosorbent assays (R&D Systems, Minneapolis, MN, USA) according to the manufacturer’s instructions.

### qRT-PCR

TRIzol^®^ reagent (Invitrogen, Thermo Fisher Scientific, Inc.) was used to extract cellular RNA according to the manufacturer’s protocol. A PrimeScript^™^ RT reagent kit (cat. no. RR037A; Takara Bio, Inc., Dalian, China) was used to synthesize cDNA. The reverse transcription reaction conditions were as follows: 37°C for 15 min, 85°C for 5 seconds, and cooling at 4°C. The qRT-PCR was conducted in accordance with our previous report [48]. A TB Green^®^ Premix Ex Taq^™^ II kit (cat. no. RR820A; Takara Bio, Inc., Dalian, China) was used for 20-μL reactions containing 10 μL 2X TB Green Premix Ex Taq II (Tli RNaseH Plus), 0.8 μL forward primer (10 μM), 0.8 μL reverse primer (10 μM), 2 μL template DNA and 6.4 μL ddH2O. The thermocycling conditions included an initial holding at 37°C for 30 min; 95°C for 1 min; 40 cycles of 95°C for 18 seconds and 60°C for 55 seconds; a final extension step at 72°C for 2.5 min; and subsequent holding at 4°C. All samples were evaluated in triplicate, and the experiments were performed three times. The primer sequences were: *IL-1β* forward 5′-TGGGAGATGGAAACATCCAG-3′ and reverse 5′-GCATTTTACTGACTGCACGG-3′; *TNF-α* forward 5′-AGAACCCCCTGGAGATAACC-3′ and reverse 5′-AAGTGCAGCAGGCAGAAGAG-3′; *IL-10* forward 5′-CTTTCACTTGCCCTCATCC-3′ and reverse 5′-ACAAACAATACGCCATTCCC-3′; *TGF-β* forward 5′-ACCCGCGTGCTAATGGTGGAC-3′ and reverse 5′-GAGCAGGAAGGGTCGGTTCAT-3′; *CC-3* forward 5′-AGCAATAAATGAATGGGCTGAG-3′ and reverse 5′-GTATGGAGAAATGGGCTGTAGG-3′; *Bax* forward 5′-GTTGCCCTCTTCTACTTTGC-3′ and reverse 5′-ATGGTCACTGTCTGCCATG-3′; *Bcl-2* forward 5′-GGTCCTCCAGTGGGTATTT-3′ and reverse 5′-TCCTCCTGAGACTGCCTTAT-3′; and *β-actin* forward 5′-TGACGTGGACATCCGCAAAG-3′ and reverse 5′-CTGGAAGGTGGACAGCGAGG-3′. *β-actin* was used as the internal control. The 2^-ΔΔCq^ method was used to measure the relative gene expression [50].

### Western blotting

Hippocampal tissues and neurons were lysed in radioimmunoprecipitation assay lysis buffer (Thermo Fisher Scientific, Inc., USA), and the protein concentration was determined using a bicinchoninic acid assay (OriGene Technologies, Inc., Beijing, China). Then, 25 μg of the extracted proteins were loaded onto 12% sodium dodecyl sulfate polyacrylamide gels, electrophoretically resolved and transferred to polyvinylidene difluoride membranes (Bio-Rad Laboratories, Inc.). The membranes were blocked with 5% skim milk for 1.5 h at room temperature. After being washed three times (3 min each) with phosphate-buffered saline (PBS) containing 0.2% Tween-20, the membranes were incubated overnight at 4°C with primary antibodies. The following primary antibodies from Abcam were used: MEK5 (ab210748; 1:1,000; rabbit polyclonal), p-MEK5 (ab254134; 1:1,000; rabbit monoclonal), ERK5 (ab196609; 1:1,000; rabbit polyclonal), p-ERK5 (ab5686; 1:1,000; rabbit polyclonal), Bax (ab32503; 1:1,000; rabbit monoclonal), Bcl-2 (ab196495; 1:1,000; rabbit polyclonal), CC-3 (ab49822; 1:1,000; rabbit polyclonal) and β-actin (ab8227; 1:1000; rabbit polyclonal). After being washed three times (5 min each) with PBS containing 0.2% Tween-20, the membranes were incubated with an IRDye^®^ 800CW goat anti-rabbit IgG secondary antibody (ab216773; 1:10,000; Abcam, USA) at room temperature for 2 h. An enhanced chemiluminescent reagent (Bio-Rad Laboratories, Inc., USA) was used to develop the signals. Protein levels were quantified using a densitometric analysis in ImageJ (Image Lab 4.1; National Institutes of Health, USA).

### TUNEL staining to detect neuronal apoptosis

Forty-eight hours after the TBI or Sham surgery, paraffin-embedded brain sections were prepared and subjected to TUNEL staining as previously described [51]. A TUNEL Apoptosis Assay Kit (cat. no. C1088, Beyotime Institute of Biotechnology, Haimen, China) was used. Briefly, 4-μm tissue sections were washed twice (5 min each) in 0.1 M PBS before being deparaffinized, rehydrated and treated with a Proteinase K working solution (10 μg/mL, pH 7.5–8.0) at 37°C for 15 min. The slices were then rinsed again in PBS and stained with a green fluorescein-labeled dUTP solution for 10 min at room temperature. Next, the sections were mounted and coverslipped with 4′,6-diamidino-2-phenylindole (DAPI; Vector Laboratories, Inc., USA) for 5 min at room temperature. A fluorescence microscope (Olympus Corporation, Tokyo, Japan) was used to detect TUNEL-positive cells that exhibited green fluorescent granules. Five fields of view were randomly selected for cell counting (200× magnification). The number of TUNEL-positive apoptotic neurons divided by the total number of DAPI-stained neurons was considered to be the percentage of apoptotic cells.

### Primary hippocampal neuron culture

The procedures for culturing primary hippocampal neurons were essentially the same as those described in a previous study [48]. Primary hippocampal neurons were obtained from Sprague-Dawley rat embryos (10 ± 2 g). Specifically, 17-day pregnant rats were anesthetized via isoflurane inhalation (4.5% induction, 2.5% maintenance), their fetuses were removed, and the pregnant rats were euthanized via cervical dislocation. The fetuses were sacrificed under deep anesthesia using CO_2_ and rapid decapitation, as described previously [52]. The scalp of each fetus was incised along the midline to expose the hemispheres of the brain. Then, the hippocampus was removed, cut into pieces and digested with trypsin (0.25%; Gibco, Thermo Fisher Scientific, Inc., USA) at 37°C for 15 min. The homogenate was filtered and centrifuged at 1,500 x *g* for 5 min at 4°C. The cells were then resuspended in medium containing 92% Neurobasal Medium, 5% fetal bovine serum (cat. no. 16140063; Gibco, Thermo Fisher Scientific, Inc., USA), 2% B27, 1% glutamate and 2 μL gentamicin. The cells were seeded at a density of 1–5 × 10^5^ cells/mL in six-well plates for 24 h. To prevent the growth of non-neuronal cells, arabinosylcytosine (10 mg/L) was added after 72 h of cultivation. Half of the volume of the culture medium was replaced every three days.

### *In vitro* TBI model

Hippocampal neurons were cultured in low-glucose medium (5.5 mM) or HG medium (25 mM) for 10 days. Then, the neurons were randomly assigned to the Control, Control+HG, TBI, TBI+HG, TBI+HG+CA-MEK5 or TBI+CA-MEK5 group. A widely accepted method was used to create the *in vitro* model of TBI [53, 54]. For the TBI, TBI+HG, TBI+HG+CA-MEK5 and TBI+CA-MEK5 groups, monolayers of primary hippocampal neurons in six-well plates were manually scratched with a 10-μL micropipette tip at 4-mm intervals. Neurons in the Control and Control+HG groups were cultured without this intervention. Then, the neurons were cultured at 37°C in a 5% CO_2_ incubator for another 24 h.

For plasmid transfection, neurons were cultured in six-well plates. The cells were transfected with a CA-MEK5 overexpression plasmid (1 μg) for 24 h using Lipofectamine^®^ 2000 (Invitrogen, Thermo Fisher Scientific, Inc.) according to the manufacturer’s instructions. The CA-MEK5 plasmid was kindly provided by Jun-ichi Abe (University of Texas MD Anderson Cancer Center, USA). For MEK5 inhibition, cells were cultured for 24 h in the presence of the selective ERK5 inhibitor BIX02189 (10 μM). The transfection efficiency and inhibition efficiency were assessed via Western blotting and qRT-PCR 24 h after the transfection. Neurons transfected with the Control plasmid (pcDNA3.1) or treated only with dimethyl sulfoxide were used as negative controls.

A CCK-8 assay (Dojindo Molecular Technologies Inc., Kumamoto, Japan) was used to determine the viability of the primary hippocampal neurons.

### Cell viability assay

Cell viability was evaluated using a CCK-8 assay according to the manufacturer’s instructions (Dojindo Molecular Technologies Inc., Kumamoto, Japan). Briefly, neurons that had undergone various treatments were seeded at a density of 1 × 10^5^ cells per well in a 96-well plate in triplicate. Subsequently, the neurons were cultured in a CO_2_ incubator at 37°C for 24 h. CCK-8 solution (10 μL) was added to each well for 1.5 h, and cell viability was measured spectrophotometrically at 450 nm.

### Immunofluorescence analysis

After being scratched with a micropipette tip, neurons were seeded at a density of 1 × 10^5^ cells per well in a six-well plate and incubated for 24 h. The neurons were fixed with 4% paraformaldehyde for 25 min and then washed twice with PBS. The cells were blocked with 10% goat serum (ab7481; Abcam) for 1 h at room temperature and then incubated at 4°C overnight with primary antibodies against p-MEK5 (cat. no. AF2551; 1:100; Beyotime, Shanghai, China) or NeuN (monoclonal; cat. no. 94403; 1:100; Cell Signaling, USA). The following day, the cells were incubated for 1 h at 37°C with an Alexa Fluor^®^ 488 goat anti-rabbit IgG secondary antibody (cat. no. ab150077; 1:1,000; Abcam) or an Alexa Fluor^®^ 647 goat anti-mouse IgG (H+L) secondary antibody. Nuclei were counterstained with DAPI for 10 min at room temperature. Fluorescence was observed with an Olympus F1000 laser scanning confocal fluorescence microscope. The fluorescence intensity was quantified using MATLAB software (MathWorks).

### Statistical analysis

Data were analyzed using SPSS 23.0 statistical software. The results are shown as the mean ± standard deviation or standard error. Each experiment was performed a minimum of three times. The results of the rotarod performance test, mNSS, forelimb placement test and MWM oriented navigation trials were analyzed using two-way mixed-model analysis of variance (ANOVA) with Sidak’s *post-hoc* test. All other data were analyzed using one-way ANOVA with Tukey’s *post-hoc* test for multiple comparisons. *P* < 0.05 (two-sided) was defined as statistically significant.
